# Evidence of Q Fever and Rickettsial Disease in Chile

**DOI:** 10.3390/tropicalmed5020099

**Published:** 2020-06-11

**Authors:** Teresa Tapia, John Stenos, Roberto Flores, Oscar Duery, Rodrigo Iglesias, Maria Fernanda Olivares, Doris Gallegos, Cristian Rosas, Heidi Wood, Johanna Acevedo, Pamela Araya, Stephen R. Graves, Juan Carlos Hormazabal

**Affiliations:** 1 Bacteriology, Sub-Department of Infectious Diseases, Department Biomedical Laboratory, Public Health Institute of Chile, Santiago 7780050, Chile; ttapia@ispch.cl (T.T.); rhflores@ispch.cl (R.F.); oduery@ispch.cl (O.D.); riglesias@ispch.cl (R.I.); paraya@ispch.cl (P.A.); 2 Australian Rickettsial Reference Laboratory, Barwon Health, Geelong, VIC 3220, Australia; JOHN.STENOS@barwonhealth.org.au (J.S.); graves.rickettsia@gmail.com (S.R.G.); 3 Departamento de Epidemiología, DIPLAS, Ministerio de Salud de Chile, Santiago 8320123, Chile; maria.olivares@minsal.cl (M.F.O.); dgallego@minsal.cl (D.G.); johanna.acevedo@minsal.cl (J.A.); 4 SEREMI de Salud Región de Los Lagos, Ministerio de Salud de Chile, Osorno 5290000, Chile; cristian.rosas@redsalud.gov.cl; 5 Zoonotic Diseases and Special Pathogens, National Microbiology Laboratory, Public Health Agency of Canada, Winnipeg, MB R3E 3R2, Canada; heidi.wood@canada.ca

**Keywords:** Q fever, *Coxiella burnetii*, rickettsiae, rickettsial infection, outbreak

## Abstract

Q fever and rickettsial diseases occur throughout the world and appear to be emergent zoonoses in Chile. The diagnosis of these diseases is currently uncommon in Chile, as their clinical presentations are non-specific and appropriate diagnostic laboratory assays are of limited availability. During a recent outbreak of undiagnosed human atypical pneumonia, we serologically investigated a series of 357 cases from three regions of southern Chile. The aim was to identify those caused by *Coxiella burnetii* and/or *Rickettsia* spp. Serological analysis was performed by ELISA and an immunofluorescence assay (IFA) for acute and convalescence sera of patients. Our results, including data from two international reference laboratories, demonstrate that 71 (20%) of the cases were Q fever, and 44 (15%) were a likely rickettsial infection, although the rickettsial species could not be confirmed by serology. This study is the first report of endemic Q fever and rickettsial disease affecting humans in Chile.

## 1. Introduction

Both coxielloses and rickettsioses are primarily zoonotic diseases with a worldwide distribution. The reservoir and mechanism of dissemination of the bacterium causing Q fever are complex and often involve unknown factors [1]. The causative agent is *Coxiella burnetii*, a small Gram-negative, pleomorphic bacterium belonging to the order Legionellales that grows intracellularly within eukaryotic cells [2,3]. The main reservoir consists of ruminant animals (goats, sheep, and cattle) in which the infection can be asymptomatic or produce reproductive disorders, such as reduced fertility, abortions, endometritis, and infertility [3,4]. The main populations at risk are abattoir and livestock workers and people working in the livestock sector (veterinarians and livestock handlers) [4,5]. Humans are infected through the inhalation of contaminated aerosols or contact with infectious urine, feces, unpasteurized milk, amniotic fluid, placental tissues, or abortion debris from infected animals [6]. *C. burnetii* has a significant capacity for long-term survival in the environment owing to a pseudosporulation process, which is an important factor in its dissemination [3]. 

The rickettsial genus is made up of four groups. The typhus group (TG), consisting of *Rickettsia typhi* and *R. prowazekii*; the spotted fever group (SFG), consisting of more than 20 characterized rickettsial species; the ancestral group (AG), consisting of *R. canadensis* and *R. belli*; and the transitional group (TRG), consisting of *R. akari*, *R. australis*, and *R. felis* [7]. The transmission of these rickettsial species is usually vector-dependent and can vary from species to species, with some species, such as *R. felis*, having multiple vectors [8]. 

The symptoms of these acute illnesses are influenza-like, including a fever, chills, headache, fatigue, malaise, and myalgia, with rashes seen rarely in Q fever, but commonly in rickettsial infections. These infections can be self-limited in duration, but are readily treated using doxycycline, which significantly reduces the illness duration [6,9].

In the presence of symptoms suggestive of Q fever, immunofluorescence assay (IFA) serology is the gold standard and the first-line diagnostic technique [6]. *C. burnetii* exists as one of two forms of antigens, called phases I and phase II, generating different patterns of the antibody response to infection. Antibodies (IgM and/or IgG) against antigens in phase II of *C. burnetii* are expressed early during acute illness [2]. In patients with chronic (persisting, focal) Q fever infection, high levels of IgG antibodies are detected against antigens in phase I. On-going, high titer anti-phase I antibodies are associated with chronic Q fever infection [1]. 

After the recognition of Q fever as a disease in Australia [10], different outbreaks of Q fever have been described around the world [6], but in South America, information has been scarce [11]. In Chile, in 1998, eight cases of Q fever were detected for the first time, in workers of the Servicio Agricola Ganadero (SAG), with the source of infection being lambs imported from Spain (unpublished data). After this event, the presence of this disease remained undetected in Chile.

In Chile, no human case of rickettsial infection has been reported. However, there is serological evidence of rickettsial infections in dogs [12] and the cat flea has been identified as the reservoir [13]. There is also evidence of rickettsial species in ticks; however the pathogenicity of these agents is currently unknown [14,15]. However, recently, cases of scrub typhus (ST), presumably caused by *Orientia tsutsugamushi,* have been described on Chiloe Island [16,17]. 

The current study describes the detection of these emergent infections and the importance of laboratory methods being available to diagnose them. The laboratory findings confirmed the presence of both *C. burnetii* and rickettsioses in Chile. The aim of the study was to serologically investigate a series of 357 suspected cases of an outbreak in three regions of southern Chile so as to identify human infection caused by *C. burnetii* and/or rickettsial agents. The establishment of laboratory diagnostic capabilities in high-risk areas and the implementation of confirmation methodologies at the National Reference Laboratory are critical steps for understanding Q fever and rickettsial infection in the Chilean population. 

## 2. Methods

### 2.1. Patients and Samples

A descriptive study was performed, including 357 human cases from an outbreak investigation conducted between July 2017 and May 2019. Patients were derived from different hospitals from three regions of Chile (La Araucanía, Los Rios, and Los Lagos). 

For the outbreak investigation, the Q fever suspect case definition included patients with hepatitis, altered liver tests, pneumonia, or atypical pneumonia and individuals of any age with fever ≥38.5 °C, headache, myalgia, cough, and associated with one or more of the following symptoms: nausea, vomiting, and diarrhea. In addition, they must have been working in hazardous locations or regions with livestock that met one or more of the following characteristics: exposure to cattle, sheep, or goats, have consumed raw animal products, or been in contact with someone who fits into either of the above categories. Rickettsial diseases have many overlapping symptoms with Q fever and thus a selection bias for rickettsial infections applied while looking for Q fever cases. One of the high-risk groups were previously healthy, young male livestock workers from Osorno province, Los Lagos region.

Of the 357 patients suspected of having Q fever, we collected 914 serum samples (acute and convalescent specimens). Of these, 312 (87.4%) cases had two or more sera available (n = 869 serum samples) and the remaining 45 (12.6%) patients had only a single serum sample. The mean age of the patients was 39.5 years, and 79.3% were men. The serum samples were stored at 4 °C or frozen at −80 °C until use. The storage and serological testing was performed at the National Reference Laboratory, Public Health Institute of Chile. Q fever is a disease that is under laboratory surveillance by the Chilean health department. Outbreak investigations, clinical data explorations, and laboratory confirmations were all performed according to the Chilean government regulations for communicable disease notification and patients’ rights and responsibilities with respect to Chilean law.

### 2.2. Serological Testing for C. burnetii and Rickettsia spp. Undertaken at International Reference Laboratories 

Acute serum samples of the first 32 cases were sent to the international reference laboratory—the National Microbiology Laboratory of Canada (NML). The serological testing for *C. burnetii* was performed using a commercial immunofluorescence assay (IFA), as per the manufacturer’s instructions (Focus diagnostics, Inc., Cypress, CA, USA). 

In addition, 47 cases (106 acute and convalescent sera) were sent to the Australian Rickettsial Reference Laboratory (ARRL), Barwon Health, Geelong, Australia. Serological testing for *C. burnetii* and rickettsiae were performed with an in-house IFA [18]. Normal cutoff titers for Q fever and rickettsial serology were 1/25 and 1/128, respectively. However, as we were screening in a previously undescribed area of infection, a more conservative approach was undertaken to enhance the specificity of the assays. Therefore, the lowest titers considered significant were raised four-fold to 1/200 for Q fever and 1/512 for rickettsial infection. The antigens used for the IFA screening consisted of *C. burnetii* phase I and II, *R. honei* (representing the spotted fever group of rickettsiae), and *R. typhi* (representing the typhus group of rickettsiae). Both IgM and IgG titers for all antigens were determined.

### 2.3. Serological Screening of C. burnetii and Definition of Criteria for the Diagnosis of Q Fever Undertaken at the National Reference Laboratory, Chile

A total of 914 serum samples from 357 patients were analyzed serologically. Both the IgM and IgG antibody response to *C. burnetii* phase I and II antigens was investigated using an IFA commercial kit, as per the manufacturer’s instructions (Focus Diagnostics, Inc., Cypress, CA, USA). Titration was carried out with dilutions according to a binary scale and an initial detection titer of 1/16 was utilized. If reactive, a doubling dilution series (1/32 to 1/1024) of each serum was prepared. Positive and negative controls were included in each test.

Acute Q fever was defined as per the suspect case definition plus the detection of IgM and IgG phase II antibodies against *C. burnetii* with a cutoff titer ≥1/128 in a single serum sample. In the case of negative serology, a request was made for follow-up serum to detect any subsequent seroconversion. In the case of a seropositive patient, a request was also made for a follow-up serum to look for any significant change in antibody titers. A four-fold increase is usually indicative of a recent infection.

Past Q fever infection was defined as having had symptoms consistent with Q fever plus IgG phase II antibody against *C. burnetii* with titers ≥1/128, and stability in paired samples (variance less than four-fold). 

### 2.4. Serological Screening for Rickettsial Infection by ELISA and IFA 

A total of 593 serum samples from 294 patients of the outbreak were analyzed for rickettsial infection, at the Public Health Institute of Chile. Of these, 269 cases were evaluated by commercial ELISA, as per the manufacturer’s instructions (Vircell, Granada, Spain), for the detection of IgM and IgG antibodies to *R. conorii*. Twenty five cases and any positive or equivocal serum by ELISA were confirmed using a commercial rickettsial IFA with *R. conorii* and *R. typhi* antigens, as per the manufacturer’s instructions (Vircell, Granada, Spain). Cutoff titers for positive rickettsial serology were IgM ≥1/384 and IgG >1/80 in single or paired samples of the patients.

## 3. Results

In July 2017, in the Los Lagos Region from Chile, an unusual outbreak of atypical pneumonia was detected that affected dairy farm workers and family members. These patients presented with one or more of the following symptoms: fever, headache, myalgia, muscle pain, cough, nausea, diarrhea, and abdominal pain. In order to determine the potential pathogen or pathogens involved in the outbreak, acute sera of the first 32 cases were sent to NML. The initial screening tested negative for viral agents (hantavirus, coronavirus (including MERS-CoV), and influenza A and B) and bacteria (*Haemophilus influenzae*, *Mycoplasma pneumoniae*, *Leptospira* spp., *Legionella, Brucella*, and *Listeria monocytogenes*). Serological results of the NML revealed five (15.6%) cases seropositive for *C. burnetii* with titer ≥1/128. This finding was the first indication of a potential etiological agent. 

With this finding, the Public Health Institute of Chile sought the assistance of the ARRL to investigate this further. Consequently, 106 serum samples from 47 patients (that fitted the case definition) were serologically analyzed for antibodies against *C. burnetii*. The results showed that 11 (23.4%) cases met the serodiagnostic criteria for Q fever infection. 

A total of 357 suspected outbreak cases from between July 2017 to May 2019 were included. Of these cases, acute serum samples (n = 357) were obtained, with an average of 26.7 days after the onset of symptoms (median of 5 days). In addition, a total of 557 convalescent serum samples were collected and included 314 second samples (average of 83.4 days, median of 60 days), 214 third samples (average of 156.1 days, median of 135 days), and 29 fourth samples, with an average of 270.3 days after the onset of symptoms (median of 206 days). All 914 specimens, both acute and convalescent sera, were analyzed by IFA. 

Of the total serum samples, 60 (6.6%) were *C. burnetii* seropositive, with IgM titers ≥1/128. Of these reactive sera, 34 (56.7%) were positive for phase II (Table 1A). Of these, 13 specimens were acute and 21 convalescent sera. The serological analysis of the IgG antibodies is shown in Table 1B. In this assay, 139 (15.2%) sera were seropositive, with titers ≥1/128. Overall, IgG phase I and II antibodies were detected in 36 (25.9%) specimens and none of the serum samples were positive for only phase I. In contrast, 103 (74.1%) specimens were seropositive for only IgG phase II (Table 1B). Of the 139 serum samples that were positive for IgG phase II, 46 corresponded to acute and 93 to convalescent sera. These findings reveal the diversity in the pattern of IgM and IgG antibodies against *C. burnetii* in our cases. There was mainly a phase II IgG response in our suspected cases. 

### 3.1. Determination of Q Fever in the Outbreak Investigation

Of the 357 patients with suspected Q fever, 71 (20%) were diagnosed by the Chilean criteria definition for this disease. A total of 31 (43.7%) patients were classified as having acute Q fever, of which 16 (51.6%) cases were seropositive, with phase II IgM and IgG antibodies (≥1/128). Of the remaining patients, 11 (35.5%) seroconverted and 4 (12.9%) had a four-fold increase of phase II IgM and/or IgG antibody titers (Table 2). 

Forty (56.3%) patients were classified as having past infection Q fever exposure (Table 2). Of these cases, 24 (60%) presented stable phase II IgG antibody levels in paired serum samples over time. Another 16 (40%) patients had antibodies to phase II and phase I IgG and/or IgM.

### 3.2. Serological Analysis of Rickettsiae in the Outbreak Investigation

In order to determine the rickettsial component of the outbreak, *R. honei*, *R. conorii*, and *R. typhi* antigens were used to examine 593 sera from 294 cases by ELISA and IFA. All the patients had suspected Q fever cases according to our case definition. This group included the 47 patients analyzed at the ARRL for *R. honei* and *R. typhi*. The remaining 247 patients were studied at our reference laboratory in Chile for *R. conorii* and *R. typhi*. 

The positive IFA results for the SFG and TG groups are shown in Table 3. The serological results revealed that 44 (15%) cases were seropositive for IgM antibodies, with titers ≥1/384 (*R. conorii*) or ≥ 1/512 (*R. honei*). Of these, 23 expressed IgM antibodies against SFG, while 20 of these patients had antibody titers against both SFG and TG. In contrast, only one case solely had antibodies to *R. typhi*; this patient was also serologically positive for acute Q fever. 

Of the tested samples, we identified 11 (25%) patients that were positive for Q fever and seropositive for IgM antibodies against the SFG with or without cross-reactive TG antibodies. Of these patients, two cases corresponded to acute Q fever and eight to past Q fever exposure. In contrast, 33 (75%) patients negative for Q fever screened were seropositive for IgM antibodies against the SFG and TG group. This analysis revealed an unexpected exposure to rickettsial infection in the outbreak investigation.

## 4. Discussion

This report reveals emerging zoonotic infections of Q fever and rickettsial disease affecting humans in Chile. Of 357 suspected patients, 71 (20%) were diagnosed by the Chilean criteria definition for Q fever, of which, 31 (43.7%) patients were classified as having acute infection. Furthermore, we detected 44 (14.7%) patients with a likely rickettsial infection, mainly as a result of the serological response to SFG rickettsiae.

In Chile, Q fever human cases were previously detected in 1998 and the infection at that time was only notifiable in animals. For humans, it became a notifiable disease in 2004, but the first human cases were not reported until July 2017. In South America, only a few epidemiological Q fever outbreaks have been published and systematic studies are lacking, probably due to the poor surveillance of the disease [2,19,20,21]. The extent of Q fever disease in South America is difficult to assess as many countries are not vigilant with disease recognition or reporting and do not have the laboratory screening methods required for disease recognition.

The gold standard for Q fever screening is considered to be the IFA, which is used as a diagnostic screening tool for both recent and past exposure. This assay is widely used due to its high sensitivity and specificity [6]. The diagnosis is dependent upon the interpretation of results using cutoff values which are defined by either the manufacturer or the local laboratories [22]. In the current study, we utilized a very conservative cutoff value of 1/128 for IgM phase II, which, in comparison, is far greater than the 1/25 cutoff utilized at the ARRL. This is likely to have caused an under-reporting of these diseases in our outbreak, but this was justifiable, in order to be certain of a high specificity, given that these infections are not currently recognized as endemic diseases in Chile. Of course, the cutoff value utilized will be evaluated and revised as more data are generated and we achieve a better understanding of Q fever disease in the Chilean population. 

The finding of serologic reactivity for the two tested SFG species, *R. honei* and *R. conorii*, and the TG member, *R. typhi*, reveals the importance of rickettsioses as emergent infections in Chile. As this diagnosis is purely serological and there is no molecular data or an isolate, the causative rickettsial species remains elusive. The causative agent of human cases of ST that have been described on Chiloe Island, southern Chile, has recently been reported to be mites found on rodent species [16,17,23]. Until this report, there was very limited information of human rickettsial disease in Chile, with one exception in the early 20th century [24]. However, there is serological evidence of rickettsial infections in domestic animals, ticks, and fleas [12,13]. A study on domestic cats identified fleas infected with *R. felis* [7,13], which may implicate this agent as the cause of the Chilean outbreak, particularly as there is serological cross-reactivity between *R. conorii* and *R felis* antigens [25]. On the other hand, the route of transmission in our current cohort is unclear, as there was no vector identified, and it is likely that infection may have occurred via a *Rickettsia* that can be transmitted via inhalation and perhaps points to a *Rickettsia* such as *R. typhi*. 

Even though we have attributed part of the outbreak to *C. burnetii* and part to *Rickettsia*, there is probably still a large number of patients with the symptoms described in this paper that have gone undiagnosed. Perhaps a larger number may have been diagnosed had we used lower cut-offs in our assays or there may be another cause. There are many zoonotic infections that are bacterial, viral, or parasitic that could have contributed to this outbreak that were not tested for and their discovery may as yet yield interesting and medically important data for the Chilean population. Moreover, other diagnostic options need to be applied, especially in hospitals in high-risk areas, including molecular diagnostics and cell culture assays for intracellular pathogens [26]. 

The absence of specific clinical features of Q fever and the complexity of the laboratory diagnosis are important and challenging tasks for the Chilean health system. There is a need for a better understanding of Q fever dynamics in the Chilean population, including epidemiological and clinical information, and a rapid laboratory method for the early detection of genuine cases. 

There is a great need to establish serology screening assays in local hospitals and a national reference laboratory for the confirmation of results and research into these local diseases. This first report of a Q fever outbreak and the serological evidence of rickettsial infections in the Chilean population is an important signal for improvement of the clinical detection and management in healthy personnel, improvement of the laboratory diagnostic capacity, and the enhancement of preventive medicine, especially in animal production areas and dairy farm workers in Chile. A recent report has confirmed the presence of *C. burnetii* in raw tank milk samples from the Los Lagos region [27]. These results support the potential public health risk of Q fever in Chile and the need for a public health strategy to control the detection and prevention of emerging zoonotic infection in livestock workers. 

## Figures and Tables

**Table 1:** Immunofluorescence assay (IFA) titers of IgM and IgG antibodies to *Coxiella burnetii* phase I and II antigens in the 914 specimens of the outbreak. Titers in bold indicate the number of specimens with elevated phase I and/or II (≥1/128).

**A) IgM Titers**									
Phase II IgM		**Phase I IgM**							
	**Titer**	**≤1/16**	**1/32**	**1/64**	**1/128**	**1/256**	**1/512**	**≥1/1024**	**Total N**
	**≤1/16**	786	24	20	**12**	**2**	**4**	0	848
	**1/32**	6	5	4	0	**1**	0	0	16
	**1/64**	4	2	3	**3**	**4**	0	0	16
	**1/128**	**2**	**4**	**3**	**3**	0	**1**	0	13
	**1/256**	**1**	0	**4**	**1**	**3**	**0**	0	9
	**1/512**	**1**	**1**	**1**	0	0	**1**	0	4
	**≥1/1024**	**4**	0	0	0	**2**	0	**2**	8
	**Total N**	804	36	35	19	12	6	2	914
**B) IgG Titers**									
Phase II IgG		**Phase I IgG**							
	**Titer**	**≤1/16**	**1/32**	**1/64**	**1/128**	**1/256**	**1/512**	**≥1/1024**	**Total N**
	**≤1/16**	607	5	3	0	0	0	0	615
	**1/32**	55	24	2	0	0	0	0	81
	**1/64**	63	10	6	0	0	0	0	79
	**1/128**	**28**	**8**	**10**	**3**	**1**	**2**	0	52
	**1/256**	**8**	**19**	**9**	**7**	**2**	**1**	0	46
	**1/512**	**1**	**9**	**8**	**3**	**2**	0	**2**	25
	**≥1/1024**	**0**	**3**	0	**3**	**7**	**2**	**1**	16
	**Total N**	762	78	38	16	12	5	3	914

**Table 2:** Patients with a serology diagnostic of Q fever. The designations I and II indicate either phase I or phase II of *C. burnetii*, respectively.

Q Fever Diagnosis	N of Patients
**Acute Q fever**	***31***
IgM II + IgG II ≥1/128	16
IgM II or/and IgG II four-fold rise in titer	4
seroconversion	11
**Past Q fever infection**	***40***
only IgG II	24
IgG II + IgG I or IgM I	12
IgG II + IgG I + IgM I	4

**Table 3:** Serological evidence of rickettsial infection in the outbreak investigation. IFA = reference method, positive cutoff of ≥ 1/512. Rickettsiae indicated by the spotted fever group (SFG) and typhus Group (TG).

Groups	Serology by IFA			Total Cases
	SFG	TG	SFG + TG	
**Positive for Rickettsiae**	23	1	20	44
negative Q fever	16	0	17	33
positive Q fever	7	1	3	11
acute Q fever	2	1	0	3
past Q fever	5	0	3	8
